# Post-partum, post-sterilization tubo-ovarian abscess caused by *Fusobacterium necrophorum*: a case report

**DOI:** 10.1186/1752-1947-6-330

**Published:** 2012-10-02

**Authors:** Chenchit Chayachinda, Amornrut Leelaporn, Pornpimol Ruangvutilert, Manopchai Thamkhantho

**Affiliations:** 1 Unit of Gynecologic Infectious Diseases and Female Sexually Transmitted Diseases, Division of Reproductive Medicine, Department of Obstetrics and Gynecology, Faculty of Medicine Siriraj Hospital, Mahidol University, 2 Prannok Road, Arun-ammarin, Bangkok-Noi, Bangkok, 10700, Thailand; 2 Department of Microbiology, Faculty of Medicine Siriraj Hospital, Mahidol University, 2 Prannok Road, Arun-ammarin, Bangkok-Noi, Bangkok, 10700, Thailand; 3 Division of Maternal-Fetal Medicine, Department of Obstetrics and Gynecology, Faculty of Medicine Siriraj Hospital, Mahidol University, 2 Prannok Road, Arun-ammarin, Bangkok-Noi, Bangkok, 10700, Thailand

**Keywords:** Post-partum, Post-sterilization, Tubo-ovarian abscess, *Fusobacterium*

## Abstract

**Introduction:**

Post-partum, post-sterilization tubo-ovarian abscess is a rare event. *Fusobacterium necrophorum* subspecies *funduliforme*, a normal flora found mainly in the oral cavity, appears to be the etiologic organism.

**Case presentation:**

In this case report, a 25-year-old Thai woman had a post-partum, post-sterilization tubo-ovarian abscess caused by the strictly anaerobic bacterium, *Fusobacterium necrophorum* subspecies *funduliforme*. Progressively severe symptoms started 3 weeks after her third vaginal delivery with a tubal sterilization on the following day. On admission, she presented with peritonitis and impending shock. An exploratory laparotomy showed a ruptured left tubo-ovarian abscess. A segment of her ileum had to be resected because of severe inflammation.

**Conclusions:**

*Fusobacterium necrophorum* subspecies *funduliforme* can be an etiologic organism of a ruptured tubo-ovarian abscess following tubal sterilization in a healthy host.

## Introduction

Tubo-ovarian abscess (TOA), characterized by an inflammatory complex mass in the pelvis, accounts for 15% of all pelvic inflammatory diseases (PIDs) [1]. A diagnosis of TOA requires the PID criteria and at least one complex pelvic mass [2]. The post-partum period appears to be the least likely time to develop a TOA because ascending infection, which is the major pathophysiology in developing PIDs in most women, rarely occurs during this phase.

Particularly in women who have had tubal sterilization (TS), the incidence of TOA is minimal because the procedure blocks communication between the genital tract and the pelvic cavity [3]. Theoretically this blockage should prevent an ascending transmission of any organisms, if present, from the genital tract proximal to the site of TS into the peritoneal cavity. This view is supported by clinical evidence that a complete or even partial occlusion of tubes appears to lessen the severity of infection when it occurs [3].

Three possible explanations of TOA after previous occluded tubes (TOAPOT) have been proposed [3]. The most likely cause is the persistent tract or re-connection between the two tubal segments. Secondly, factors related to the operative procedure are possible. Lastly, systemic factors such as a hematogenous or lymphatic bacterial spread with a compromised immunological status of the patient are possible. In this case report, a 25-year-old Thai woman had a TOAPOT caused by *Fusobacterium necrophorum* subspecies *funduliforme*.

## Case presentation

A 25-year-old Thai woman (*gravida 3, para 3*) had her third uneventful vaginal delivery followed by TS by modified Pomeroy technique on the following day. She experienced a persistent low-grade fever and abdominal pain that began in the third post-partum week and deteriorated over time. Her medical history was unremarkable. She denied any history of intravenous drug abuse, smoking, alcohol intake, or abdominal trauma. She reported being monogamous. Her partner was healthy and denied any history of sexually transmitted infections. Her previous obstetric history was unremarkable at 8 years and 5 years prior to this delivery. The vaginal delivery of her third baby took place 2 hours after a spontaneous rupture of membranes and progressed uneventfully. Post-partum TS was performed by request without any remarkable findings. She recovered well and was discharged with her baby from the hospital 3 days later.

With a persistent and progressive fever for 1 week, she was admitted to the hospital. On admission, she looked very sick. A physical examination showed a high-grade fever (39°C), tachycardia (pulse rate of 112 beats per minute), tachypnea (respiratory rate of 26 per minute), and a blood pressure of 110/60mmHg. An abdominal examination showed a small transverse subumbilical incision scar (from the TS) without any signs of wound infection. A 16-week, pregnancy-sized, non-mobile pelvic mass was found on the left side of her pelvis. The mass and other areas of her pelvis and abdomen were tender, suggesting peritonitis. A pelvic examination revealed a large amount of purulent vaginal discharge, a marked degree of pain elicited upon cervical excitation, a bulging cul-de-sac, and the tender pelvic mass as described in the abdominal examination findings. The results of the rest of the physical examination were unremarkable.

Ultrasonograms showed a uterus of 10.1×6.2×5.1cm in size and a smooth thin endometrial lining. A multiloculated mass of 6.2×8.2×10.9cm in size was seen over the left adnexal region. The right adnexal region was unremarkable. Approximately 50mL of free fluid was noted in the cul-de-sac. Laboratory testing revealed a hematocrit level of 32.2%, a white blood cell count of 33,170/mm^3^ (90.2% were polymorphonuclear neutrophils), and a platelet count of 422,000/mm^3^. Levels of blood urea nitrogen and creatinine were 6.2 and 0.5mg/dL, respectively. Blood electrolytes were as follows: sodium 138mmol/L, potassium 3.4mmol/L, chloride 106mmol/L, and bicarbonate 22mmol/L. A slightly prolonged coagulogram was found: a prothrombin time of 15.3 (10 to 13) seconds and a partial thromboplastin time of 34.7 (21 to 30) seconds. The results of a liver function test were normal. The results of a cervical swab and blood culture were negative.

The clinical diagnosis was a ruptured TOA. After counseling for a diagnosis and a plan for an urgent exploratory laparotomy, intravenous clindamycin 2700mg per day and gentamicin 240mg per day were initiated. Fluid resuscitation and preparation of blood components were executed.

**Figure 1:**
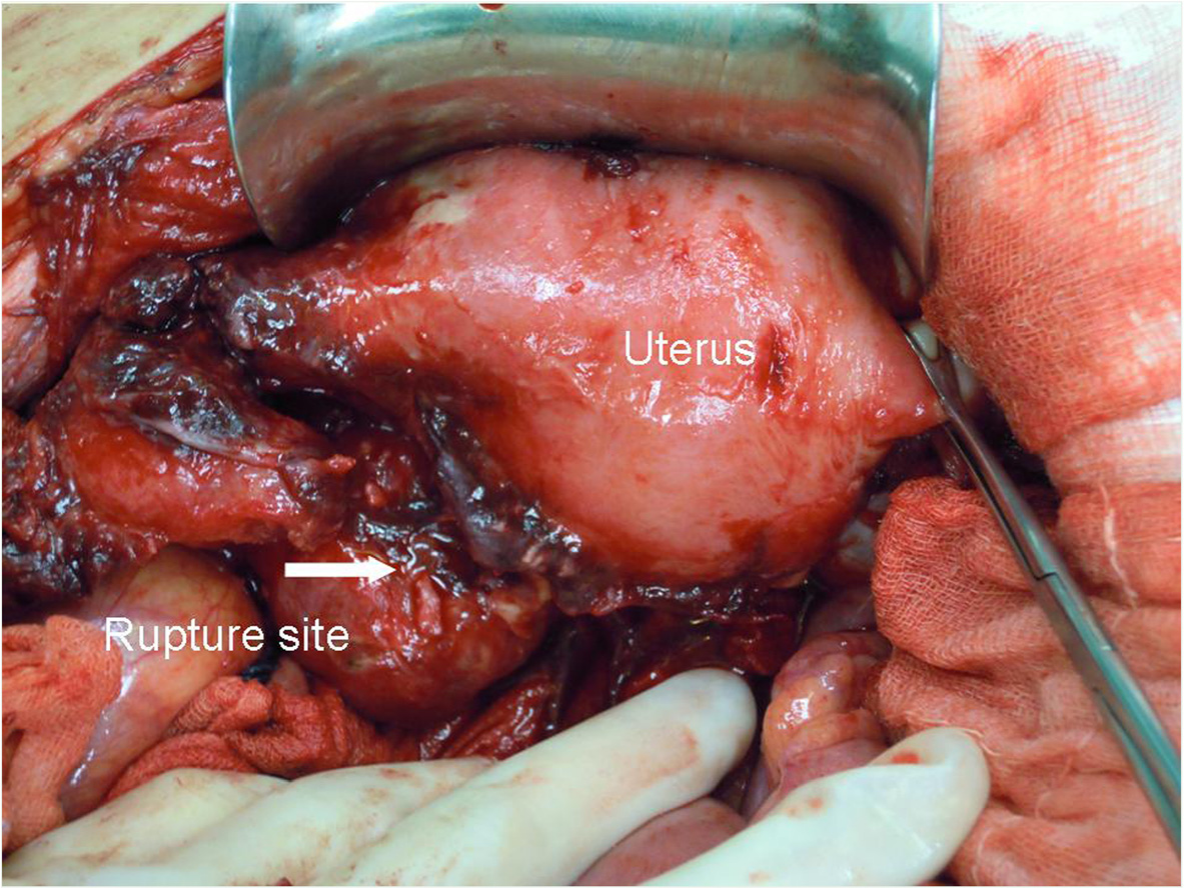
During the operation, a ruptured left tubo-ovarian abscess is found.

**Figure 2:**
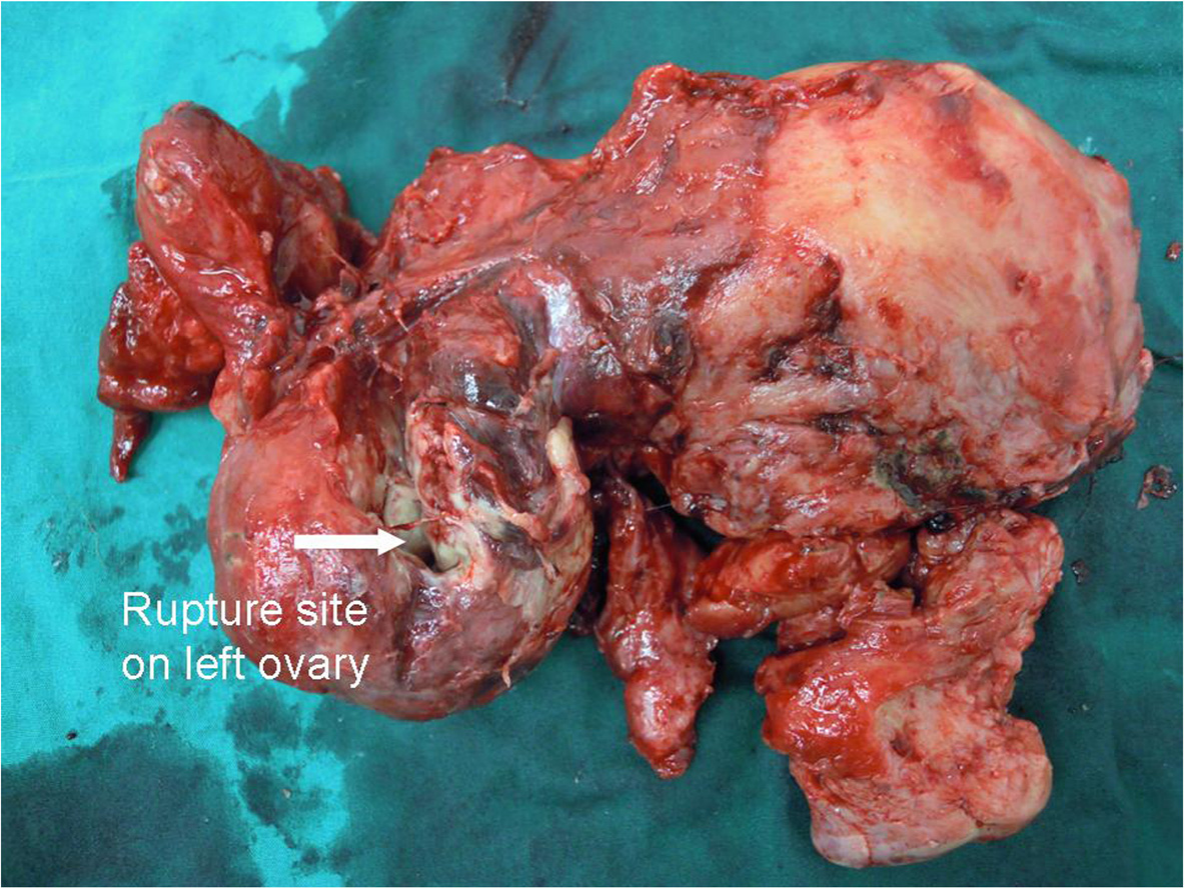
A uterus specimen and a ruptured left tubo-ovarian abscess.

**Figure 3:**
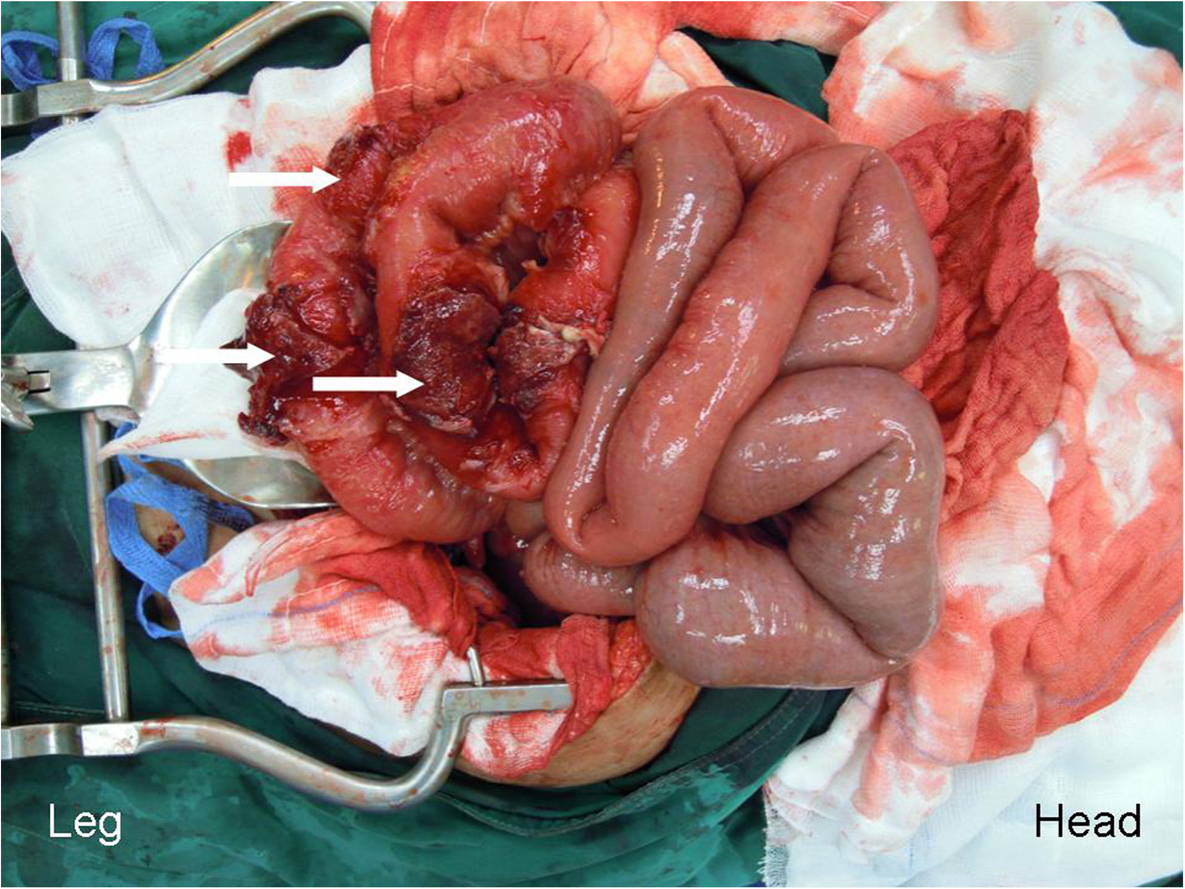
The severely inflamed ileum and the attaching sites to the ruptured tubo-ovarian abscess (arrows).

**Figure 4:**
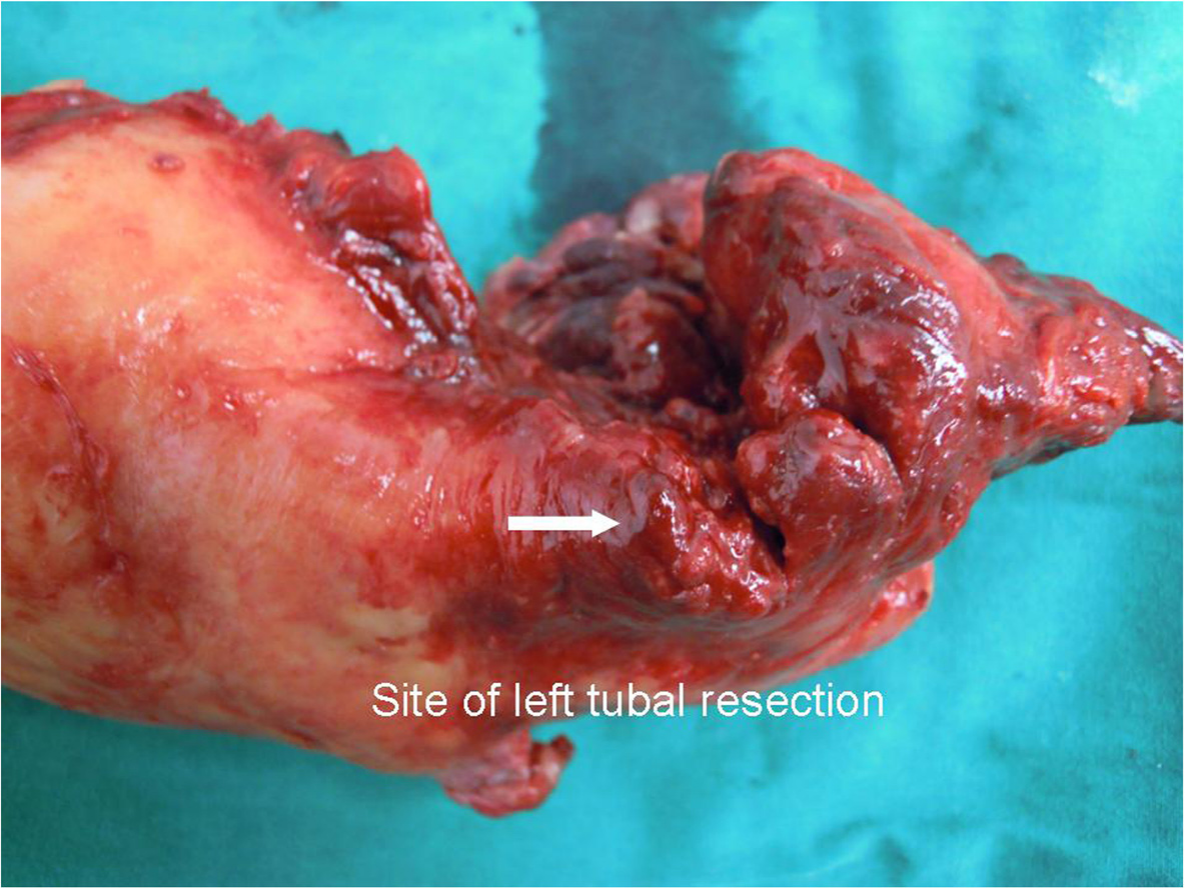
**Suture material for tubal sterilization is absent.** Severe infection is shown.

The operative findings showed 50mL of bloody purulent intraperitoneal fluid. A left tubo-ovarian complex (Figures 1 and 2) of 7×7cm in size was seen. The mass contained 30mL of malodorous pus and had a 2cm rupture site on its posterior surface (Figures 1 and 2). The rupture site was walled off by a 30cm loop of severely inflamed ileum (Figure 3). The two free ends of proximal and distal parts of the left fallopian tubes were evidenced (Figure 4) without residual pieces of chromic catgut used for the sterilization. The uterus was markedly inflamed. The right adnexal region appeared grossly normal.

A total hysterectomy with left salpingo-oophorectomy, ileal resection with end-to-end anastomosis, abdominal toileting, and Penrose drain placement were performed. The operation took 4 hours, and the estimated blood loss was 2000mL. A Gram stain of pus from the abdominal cavity demonstrated numerous polymorphonuclear cells and numerous coccoid pleomorphic Gram-negative rods. An abdominal fluid culture revealed no growth, whereas the pus culture showed *Fusobacterium necrophorum*. The organism was identified as *F. necrophorum* subsp. *funduliforme* by microscopic examination, biochemical characteristics, and 16S ribosomal deoxyribonucleic acid (rDNA) sequencing. The isolate was designated as SIRD333. The 658 base-pair fragment of SIRD333 16S rDNA was submitted to GenBank (accession number JX103157). The surgical pathology showed chronic endometritis, TOA, and ileitis.

Our patient recovered well after the operation and had further treatment with intravenous antibiotics (clindamycin and gentamicin) until being clinically stable for 48 hours (4 days of intravenous antibiotics in total). She was discharged on the 10th post-operative day. She continued oral antibiotics (clindamycin) for six more weeks. She was clinically well during all of her follow-up visits.

## Discussion

To the best of our knowledge, this is the first case report of *F. necrophorum* as the etiologic organism of TOA developing during the post-partum period in a patient who had undergone TS. At our institution, where more than 5000 TS procedures are performed annually by using the modified Pomeroy technique, post-sterilization TOAs are rarely encountered.

Time intervals from tubal occlusion to TOAPOT had been reported to range from as early as 36 hours to up to 12 years [3]. Our patient presented with a gradual onset of systemic inflammatory response syndrome (tachycardia, tachypnea, high-grade fever, and high count of white blood cells) and peritonitis starting from the third week after sterilization. Since post-partum sexual intercourse had not been engaged in and the history was unremarkable, systemic factors such as hematogenous or lymphatic bacterial spread [3] or pelvic infection from adjacent organs [4] appeared to be the most probable mechanisms. The partially resected fallopian tubes might promote favorable conditions for anaerobic invasion and growth [4].

*Fusobacterium* is a strictly anaerobic, Gram-negative bacillus. Among this group, *F. necrophorum* is the most virulent species. This species has been divided into two subspecies: *F. necrophorum* subsp. *funduliforme* and *F. necrophorum* subsp. *necrophorum*. The former subspecies is usually associated with human infection, whereas the latter is usually associated with other mammals [5].

*F. necrophorum* subsp. *funduliforme* normally colonizes in the oral cavity [6] but can be found in abdomino-pelvic viscera and the female genital tract [7]. It can cause infection in a variety of human viscera such as the head and neck, the respiratory tract, the abdomen and pelvis, and the female genital tract [8]. Transient bacteremia of *F. necrophorum* has been found to be related to preterm labor, chorioamnionitis, dead fetus *in utero*, and TOA [8]. As our patient denied a history of a sore throat in the previous 4 weeks, the source of the etiologic organism was likely to have been from the adjacent organs or the genital tract itself. The breakage of the fallopian tubes from the TS was the most likely pathogenesis because the procedure might allow the spreading of organisms into the peritoneal cavity.

In general, TOA responds well to the empirical antibiotic regimen recommended by the Centers for Disease Control and Prevention [2]. However, approximately 25% of TOA cases need surgical intervention or drainage [4]. Four principal indications for laparotomy/laparoscopy among these patients are suspicion of a surgical emergency (rupture of the abscess or organs), unsuccessful drainage of the abscess, poor response to treatment with drainage and antibiotics, and uncertainty about the diagnosis [4]. Kuo *et al*. [9] found that, among multiparous patients, hysterectomies were more commonly performed. The reported case required an exploratory laparotomy because of the suspicion of a ruptured TOA. The hysterectomy had to be performed in order to get rid of all sources of infection.

## Conclusions

*F. necrophorum* subsp. *funduliforme* can be an etiologic organism of a ruptured TOA following TS in a healthy host. Early detection and early treatment by a multidisciplinary care team can significantly reduce the maternal mortality and morbidity.

## Consent

Written informed consent was obtained from the patient for publication of this case report and any accompanying images. A copy of the written consent is available for review by the Editor-in-Chief of this journal*.*

## Abbreviations

PID: pelvic inflammatory disease; rDNA: ribosomal deoxyribonucleic acid; TOA: tubo-ovarian abscess; TOAPOT: tubo-ovarian abscess after previous occluded tubes; TS: tubal sterilization.

## Competing interests

The authors declare that they have no competing interests.

## Authors’ contributions

CC helped to review the case and complete the case summary and reviewed the literature. PR and MT helped to review the case and complete the case summary. AL performed the microbiological examination. All authors read and approved the final manuscript.

## References

[B1] Landers D Sweet R Tubo-ovarian abscess: contemporary approach to management Rev Infect Dis 1983 5 876 884 10.1093/clinids/5.5.876 6635426

[B2] Department of Health and Human Services Centers for disease control and prevention: pelvic inflammatory disease MMWR 2010 59 RR-12 63 67

[B3] Levgur M Duvivier R Pelvic inflammatory disease after tubal sterilization: a review Obstet Gynecol Surv 2000 55 41 50 10.1097/00006254-200001000-00022 10639678

[B4] Granberg S Gjelland K Ekerhovd E The management of pelvic abscess Best Pract Res Clin Obstet Gynaecol 2009 23 667 678 10.1016/j.bpobgyn.2009.01.010 19230781

[B5] Brazier JS Human infections with *Fusobacterium necrophorum* Anaerobe 2006 12 165 172 10.1016/j.anaerobe.2005.11.003 16962962

[B6] Han YW Fardini Y Chen C Iacampo KG Peraino VA Shamonki JM Redline RW Term stillbirth caused by oral *Fusobacterium nucleatum* Obstet Gynecol 2010 115 2 Pt 2 442 445 20093874 10.1097/AOG.0b013e3181cb9955 PMC3004155

[B7] Alston J Necrobacillosis in Great Britain Br Med J 1955 2 1524 1528 10.1136/bmj.2.4955.1524 13269907 PMC1981472

[B8] Huggan PJ Murdoch DR Fusobacterial infections: clinical spectrum and incidence of invasive disease J Infect 2008 57 283 289 10.1016/j.jinf.2008.07.016 18805588

[B9] Kuo CF Tsai SY Liu TC Lin CC Liu CP Lee CM Clinical characteristics and treatment outcomes of patients with tubo-ovarian abscess at a tertiary care hospital in Northern Taiwan J Microbiol Immunol Infect 2012 45 58 64 10.1016/j.jmii.2011.09.021 22154676

